# Pelvic Floor Health and Urinary Incontinence in Female Soccer Players: A Comparative Analysis Between Professionals and Physically Active Women: A Cross-Sectional Descriptive Protocol

**DOI:** 10.3390/diagnostics15151881

**Published:** 2025-07-26

**Authors:** Julia M. Sebastian-Rico, María Jesús Muñoz-Fernández, Luis Manuel Martínez-Aranda, África Calvo-Lluch, Manuel Ortega-Becerra

**Affiliations:** 1 Department of Sports and Computer Sciences, Faculty of Sports Sciences, Universidad Pablo de Olavide, 41013 Seville, Spain; jmsebric@alu.upo.es (J.M.S.-R.); acalllu@upo.es (Á.C.-L.); maortbec@upo.es (M.O.-B.); 2 Department of Physiotherapy, Francisco Maldonado University School, 41640 Osuna, Seville, Spain; mariajmf@euosuna.org; 3 CTS 1110, UMSS Research Group, University of Seville, 41640 Seville, Spain; 4 Science-Based Training Research Group (SEJ-680), Physical Performance and Sports Research Center, Universidad Pablo de Olavide, 41013 Seville, Spain

**Keywords:** athletic women, exercise-induced urinary leakage, pelvic dysfunction, sports gynecology, urinary symptoms

## Abstract

**Background/Objectives:** Urinary incontinence (UI), defined as the involuntary loss of urine, is common among female athletes. As more women engage in competitive sports, numerous studies have explored UI in young, nulliparous, and physically active women. The objectives of this study were (i) to analyze the prevalence, severity, and characteristics of UI in professional nulliparous female soccer players and (ii) to compare the status of the pelvic floor muscles (PFMs) between professional soccer players and physically active young women. **Methods:** This descriptive cross-sectional study included professional soccer players (*n* = 18) and physically active women (*n* = 14). UI was assessed using the ICIQ-SF questionnaire, and PFM function was evaluated through intracavitary examination using the PERFECT method. Additional data were collected on body composition and on urinary, bowel, and sexual health. **Results:** UI affected 35.7% of physically active women and 50% of professional soccer players. Stress urinary incontinence (SUI) was the most common type, present in 100% of affected soccer players and 60% of affected active women. The severity of UI was mostly mild, with no significant differences between groups. PFM assessment revealed deficiencies in control, relaxation, endurance, and rapid contractions, as well as difficulties performing an effective perineal locking (PL) maneuver during increased intra-abdominal pressure. **Conclusions:** These findings highlight the need for targeted programs focused on strengthening and educating athletes about their PFMs, aiming to prevent UI and improve both performance and quality of life. The study reinforces the importance of preventive strategies for pelvic floor health in sports.

## 1. Introduction

The study of women’s soccer performance has traditionally focused on analyzing the physical and physiological characteristics that optimize fitness in relation to the sport’s technical and tactical demands [1]. Consequently, research has also examined functional balance as a means of reducing injury profiles and improving the overall health of female soccer players [2,3,4,5]. In this context, the pelvic floor (PF) plays a crucial role in physical activity and training. In addition to supporting the pelvic organs, it also serves an anticipatory and reflexive function in response to increased intra-abdominal pressure [6].

Several studies have shown that during activities such as running and jumping, the pelvic floor muscles (PFMs) undergo a lengthening process and activate in anticipation of ground contact. This feed-forward activation mechanism prepares the PFM to efficiently absorb and manage impact forces. Upon ground contact, a reflexive PFM response is triggered, contributing to pelvic stabilization, tissue protection, and the maintenance of functional control during dynamic movement [7,8,9,10].

When the pelvic floor’s anticipatory contraction is absent, athletes may experience urinary incontinence (UI). UI, defined as the involuntary loss of urine that can be objectively verified [11], is a common condition among female athletes [12,13,14,15]. As women’s participation in competitive sports continues to grow, numerous studies have investigated the prevalence of UI in young, nulliparous, and physically active women, with higher incidence rates reported among those participating in high-impact sports [13,16]. Disciplines such as volleyball, gymnastics, basketball, and soccer—which involve repetitive jumping and abrupt increases in intra-abdominal pressure—have been identified as risk factors for the development of stress urinary incontinence (SUI) in nulliparous women [17]. In this scenario, PFM weakness or fatigue plays a key role in the involuntary loss of urine during intense physical activity [12].

Moreover, there is evidence linking increased intra-abdominal pressure during certain exercises to muscular imbalances that heighten the risk of UI in athletes who have not undergone adequate PFM training [18,19,20]. These findings underscore the importance of physical training programs specifically tailored for women to enhance both health status and athletic performance. In soccer, few studies have explored the role of the PFMs in athletic performance at the competitive level [14]. Most existing research has focused on lower limb injuries, particularly the high incidence of anterior cruciate ligament (ACL) injuries and hamstring strains [21].

The pelvic floor is one of the few physiological systems for which the benefits of physical activity have been questioned, sparking significant scientific debate [19]. This controversy revolves around two main theories [22]. The first suggests that the PFM is naturally exercised alongside other muscle groups during sports participation. According to this view, physically active women should exhibit stronger, more functional, and more efficient pelvic floors, as general training promotes improvements in strength, endurance, and muscular coordination—potentially enhancing pelvic stability and organ support. In contrast, the second theory argues that repeated mechanical overload of the PFM in female athletes, especially in high-impact or repetitive-effort sports, may lead to changes in pelvic connective tissue. Chronic stress on these tissues could result in structural weakening, reduced function, and a heightened risk of dysfunctions such as stress urinary incontinence, fecal incontinence, and in some cases, dyspareunia. These dysfunctions may stem from reduced elasticity and responsiveness of both connective and muscular pelvic tissues, diminishing support effectiveness and negatively impacting quality of life [19,23,24,25,26].

Findings from the above-mentioned studies suggest that, even without the perineal trauma typically associated with childbirth, nulliparous female athletes exposed to repetitive and intense physical exertion display significant rates of UI [18,19,27]. This condition not only affects athletic performance but also compromises physical and psychological well-being [28]. Additionally, limited awareness regarding pelvic floor health within this population presents a major barrier to implementing preventive training strategies focused on the pelvic floor muscles (PFMs).

In this context, the present study aims to (i) analyze the prevalence, severity, and characteristics of UI in professional nulliparous female soccer players and (ii) compare the condition of the PFM between professional female soccer players and physically active young women. The findings are intended to contribute to the development of targeted preventive training strategies for UI, promoting pelvic floor balance both in the personal lives and sporting careers of professional female soccer players.

## 2. Materials and Methods

### 2.1. Study Design

A descriptive cross-sectional study was conducted involving female soccer players from the Spanish professional leagues and physically active young women. The research was carried out in accordance with the recommendations of the World Medical Association’s Declaration of Helsinki, the Council of Europe’s Convention on Human Rights and Biomedicine, the UNESCO Universal Declaration on the Human Genome and Human Rights, and the Convention for the Protection of Human Rights and Dignity of the Human Being with regard to the Application of Biology and Medicine [29], as well as the General Data Protection Regulation (GDPR) and the Spanish Organic Law 3/2018 [30]. The study was approved by the Ethics Committee of Pablo de Olavide University (Seville, Spain), under registration code 24/5-1.

### 2.2. Participants

The study sample consisted of professional female soccer players who were active during the 2024–2025 season and physically active young women. Soccer players were contacted through their clubs, while physically active women were recruited from several universities. The total sample (*n* = 32) was divided into two groups: 18 professional soccer players formed the “Soccer Player Group” (SPG) and 14 young women formed the “Physically Active Women Group” (PAWG).

Inclusion criteria were professional female soccer players and physically active women over 18 years of age who had never been pregnant or given birth. Exclusion criteria included history of pelvic surgery, body mass index (BMI) over 25 kg/m^2^, chronic respiratory diseases, pelvic organ prolapses (POPs), or neurological conditions that could interfere with understanding the questionnaires. All participants were informed of the study objectives, instructions for completing the questionnaires, the confidentiality of their personal data, and their right to access the generated results upon completion of the study.

### 2.3. Procedure

Anthropometric variables, including height and weight, as well as body composition parameters (percentage of fat and lean mass), were assessed using a stadiometer (Tallímetro 216, Seca, Cantabria, Spain, 3.5–230 cm) and a segmental multifrequency DSM-BIA body composition analyzer (InBody120, InBody, Barcelona, Spain; 20 and 100 kHz) [31]. Participants completed the ICIQ-SF questionnaire [32,33] to assess urinary incontinence (UI), developed by the International Continence Society (ICS) and translated and validated into Spanish [34,35]. UI was classified on the basis of self-diagnostic items of the ICIQ-SF. SUI was diagnosed if the patient experienced urine leakage during coughing, sneezing, or physical activity; UUI was diagnosed if the patient experienced urine leakage before she could reach the toilet; and MUI was diagnosed if the patient had both SUI and UUI.

The ICIQ-SF was used to assess the severity and impact of urinary incontinence. This instrument has demonstrated strong psychometric properties [32], including good internal consistency (Cronbach’s alpha ranging from 0.79 to 0.93) and test–retest reliability, and is widely validated for both clinical and research purposes in diverse populations.

A personal anamnesis was also conducted, including training duration and type. Additional questions addressed urinary, bowel, and sexual health, including frequency of urination and defecation, completeness of emptying, and the presence of pain during defecation and/or sexual intercourse.

Pelvic floor muscle (PFM) assessment was performed through a physical examination by an experienced physiotherapist specialized in pelvic floor dysfunctions, following the PERFECT scheme [34] (P = Power; E = Endurance; R = Repetitions of endurance; F = Fast contractions). “Power” was assessed through a maximal voluntary contraction (MVC). “Endurance” involved holding the MVC for up to 15 s. “Repetitions” were the number of times the participant could sustain that contraction, up to a maximum of 20. “Fast contractions” referred to how many rapid, forceful contractions could be completed within 60 s while maintaining initial power. Muscle pressure was measured using a manometric perineometer (EPI-NO^®^ Delphine, Tecsana GmbH, München, Germany, ±5 cmH_2_O) [36].

The physical examination took place in a private room with a treatment table. Disposable gloves and water-based lubricant were used. Vaginal bidigital palpation was performed with participants in the supine position with knees flexed at 45°. Participants were instructed to “contract the PFM as if trying to stop the flow of urine,” lifting the muscles upward and inward. They were also instructed to inhale during PFM relaxation and exhale during contraction. Care was taken to avoid recruitment of accessory muscles, including the abdominals, gluteals, or legs [37]. A cough test was performed both with anticipatory perineal contraction (the “knack”) to assess voluntary PFM control [38], and without anticipatory instruction, to assess the involuntary response during effort [39].

The procedure for each participant included the following steps: (1) signing informed consent; (2) anthropometric assessment (height, weight, fat mass, and lean mass); (3) completion of the ICIQ-SF questionnaire; (4) a personal interview including anamnesis, training details, and urinary, bowel, and sexual function; and (5) intracavitary vaginal bidigital examination (PFM assessment using the PERFECT method and pressure measurement via perineometer).

Participants were given the following instructions during evaluation: to contract the PFM as if trying to stop urination or fit into tighter pants—lifting the muscles upward and inward; to breathe by relaxing during inhalation and contracting during exhalation; and to avoid engaging auxiliary muscles (abdomen, gluteals, or legs) during pelvic floor contractions.

### 2.4. Statistical Analysis

Descriptive statistics were calculated using the mean (M) and standard deviation (SD). Where applicable, results are presented as percentages. Statistical significance was set at *p* ≤ 0.05. The distribution of each variable was tested using the Shapiro–Wilk normality test, and homogeneity of variance was evaluated using Levene’s test. When data did not meet normality assumptions, non-parametric tests were applied, specifically the Mann–Whitney U test to compare differences between groups. Additionally, chi-square tests were used to assess associations between categorical variables, such as UI prevalence across groups.

To analyze relationships between variables, Spearman’s rank correlation coefficient (ρ) was used due to its appropriateness for non-parametric data. Correlation strength was interpreted as follows: 0.00–0.10 = negligible; 0.10–0.30 = weak; 0.30–0.50 = moderate; 0.50–0.70 = strong; 0.70–0.90 = very strong; and >0.90 = nearly perfect to perfect correlation. All statistical analyses were performed using SPSS software (version 21.0, SPSS Inc., Chicago, IL, USA) and the free statistical software JASP (version 0.9.2, University of Amsterdam, Amsterdam, The Netherlands).

## 3. Results

Table 1 presents the descriptive variables of the participants (*n* = 32), divided into the soccer players group (SPG; *n* = 18) and the physically active women’s group (PAWG; *n* = 14), including age, weight, BMI, body fat percentage, and weekly training volume.

The analysis of the ICIQ-SF questionnaire scores is presented in Table 2. The Physically Active Women’s Group (PAWG) showed an average score of 3.00 ± 5.2 (95% CI: 0.04–6.0), while the soccer players group (SPG) had a mean score of 2.4 ± 3.0 (95% CI: 0.9–3.9). The table also presents the classification of UI severity and type. No significant differences were found between the groups. Notably, no cases of very severe UI (up to 18 points) were reported in either group. A weak, non-significant correlation was found between weekly training volume (in minutes) and the ICIQ-SF score (ρ = 0.34; *p* = 0.17).

**Figure 1:**
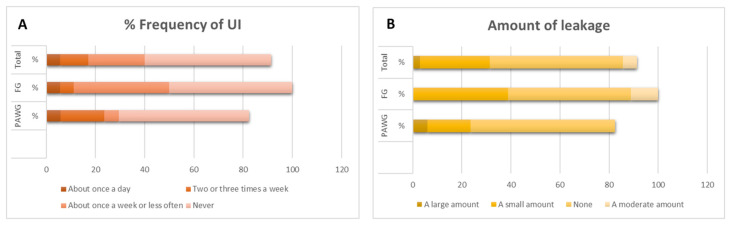
(**A**) Percentage of frequencies of UI. (**B**) Amount of leakage.

Additionally, data were collected on the frequency of urine loss (Figure 1A), the perceived amount of leakage (Figure 1B), and the impact of urinary incontinence on quality of life. Regarding this latter aspect, the PAWG reported a mean score of 1.14 ± 2.5, while the SPG showed a mean of 0.5 ± 1.2. These results suggest that, although the overall impact of UI was low in both groups, participants in the PAWG experienced a slightly greater impact compared to those in the SPG.

Table 3 presents the results of the PERFECT method assessment and physical examination tests. No significant differences were observed between the two groups.

Regarding the perception of urinary incontinence type, 88.9% (*n* = 16) of participants in the Physically Active Women’s Group (PAWG) reported no symptoms of urinary urgency, compared to 57.1% (*n* = 8) in the soccer players group (SPG). This difference was statistically significant (*p* = 0.04). Complete bladder emptying was reported by 92.9% (*n* = 13) of the SPG and 88.9% (*n* = 16) of the PAWG, with no significant difference (*p* = 0.70). Nocturia was reported at similar rates in both groups: 28.6% (*n* = 4) in the SPG and 27.8% (*n* = 5) in the PAWG (*p* = 0.96).

Daily defecation frequency was more common in the SPG (71.4%, *n* = 10) than in the PAWG (38.9%, *n* = 11), although this difference was not statistically significant (*p* = 0.54). Difficulty with defecation was more frequently reported among soccer players (42.9%, *n* = 6) compared to physically active women (22.2%, *n* = 4), but the difference did not reach statistical significance (*p* = 0.21). A feeling of complete rectal emptying was reported by 88.9% (*n* = 16) of the PAWG and 64.3% (*n* = 9) of the SPG, indicating a non-significant trend (*p* = 0.09). Pain during defecation was reported by 14.3% (*n* = 2) of the SPG and 11.1% (*n* = 2) of the PAWG, with no significant difference (*p* = 0.78).

Pain during sexual intercourse was reported by 21.4% (*n* = 3) of the SPG and 16.7% (*n* = 3) of the PAWG, with no statistically significant difference (*p* = 0.73).

## 4. Discussion

The objective of this study was to determine the prevalence, severity, and type of urinary incontinence (UI), as well as to assess participants’ pelvic floor muscle (PFM) function. Results showed that UI was present in 35.7% of young, nulliparous, physically active women and 50% of professional soccer players in our sample. Stress urinary incontinence (SUI) was the most common type in both groups—100% in the soccer players group (SPG) and 60% in the Physically Active Women’s Group (PAWG). In terms of severity, most cases in both groups were classified as mild, with no statistically significant differences. PFM evaluation indicated limited capacity to maintain maximal voluntary contraction (MVC), along with deficiencies in relaxation, endurance, and rapid contraction abilities. Notably, only 3.1% (*n* = 1) of participants were able to perform a perineal lock (the “knack” maneuver) during an increase in intra-abdominal pressure.

These findings are consistent with those reported by Mendoza et al. [40], who found UI prevalence rates of 53.8% among nulliparous female athletes and 35.3% in a control group. In our study, UI frequency (50% in the SPG, 35.7% in the PAWG), as assessed by the ICIQ-SF questionnaire, complements previous findings in young, nulliparous athletic populations. A recent review reported UI prevalence ranging from 5.75% to 80% among young athletes, depending on the sport [41]. Sports with higher prevalence include those involving significant PFM impact and elevated intra-abdominal pressure, such as trampolining, volleyball, artistic gymnastics, soccer, handball, and judo.

SUI was the most prevalent type in both groups (100% SPG, 60% PAWG). Soccer players frequently report urinary leakage during training or competition. Two main hypotheses have been proposed to explain this phenomenon: (i) athletes may develop stronger PFMs, or (ii) they may overload and overstretch their PFMs, leading to weakening. In both cases, continence-related dysfunction appears, suggesting that chronic overload could damage connective tissues. A cross-sectional study of 503 athletes reported a 14.3% UI prevalence, with SUI being the most common (13.5%) [42]. Thus, SUI seems to result from a combination of repeated intra-abdominal pressure increases and weakened PFMs in nulliparous athletes.

These findings highlight the importance of identifying UI in female athletes during their active years. A study conducted Bo & Sundgot-Borgen [43] with former athletes concluded that, although the prevalence of UI was not higher in the group of former athletes compared to the control group—suggesting that past participation in competitive sports is not necessarily associated with an increased risk of UI—having experienced UI episodes during the athletic career may be a predictive factor for its occurrence later in life. This underscores the need to monitor and address UI from an early age, particularly in physically active women, as early symptoms could serve as indicators of long-term risk.

In our study, the soccer players trained an average of 550 min per week, compared to 296.1 min per week of varied physical activity (walking, gym workouts, aerobic exercise, dancing, etc.) in the PAWG. Soccer is classified as a high-impact sport [11], and this training volume may produce imbalances in the load the PFMs must resist to maintain continence. Almeida et al. [44] concluded that female athletes have three times greater risk of UI due to excessive PFM loading, which can cause structural damage to muscles, fascia, and ligaments involved in continence [19]. The prevalence of UI observed in our sample aligns with studies linking athletic training load and UI incidence [42,45,46]. Although no statistically significant association was found between training volume and UI in this study (*p* = 0.17), the small and heterogeneous sample may have limited the statistical power to detect subtle relationships. Future studies with larger, more homogeneous samples are necessary to clarify this relationship. Nygaard et al. [47] proposed the concept of “threshold continence,” referring to the point at which PFMs can maintain continence under physical stress. This may explain UI in young, nulliparous women without anatomical or neurological impairments. Our findings emphasize the need to evaluate how weekly training load may affect PFM function and exacerbate UI symptoms in athletes.

PFM assessment showed no significant differences between groups across measured variables. Most participants demonstrated the ability to contract both the transverse abdominal muscle and the PFM (78.6% SPG, 77.8% PAWG). However, 78.6% of the SPG and 66.7% of the PAWG could not sustain MVC of the PFMs. Furthermore, none of the soccer players were able to involuntarily contract the PFMs during a cough, and only 3.1% (*n* = 1) in the PAWG achieved this. These findings reveal limited involuntary PFM control during increases in intra-abdominal pressure, highlighting the importance of targeted training interventions—even in physically trained women—to improve PFM responsiveness during sport and daily activities [19,48,49].

When voluntary contraction was requested, 50% of the SPG and 27.8% of the PAWG were able to properly control their PFMs. However, 35.7% of the SPG and 38.9% of the PAWG were unable to relax their PFMs. A recent study [50] of 44 young, nulliparous women (18–30 years) found insufficient knowledge about the PFM and its function, leading to poor control of muscle strength, endurance, and rapid contraction ability. In the present study, the PERFECT method revealed similar results in transverse abdominal muscle (*p* = 0.96) and PFM contraction (*p* = 0.96) between the two groups. A slight trend toward better endurance and relaxation was observed in the SPG, though not statistically significant (*p* = 0.46; *p* = 0.85). These results suggest the value of integrating PFM assessment and training into initial screenings and regular training protocols in professional soccer. The lack of rapid and dynamic contractions among most participants further supports the need to improve pelvic floor training. Enhancing PFM knowledge and control is essential, even in women with existing muscular capacity [51].

A significant difference was observed in the ability to reach the toilet without urinary leakage, with better results among soccer players (88.9%) compared to physically active women (57.1%) (*p* = 0.04), possibly reflecting improved PFM control among athletes in this specific context. However, no significant differences were found in variables related to bowel function (defecation frequency, perception of complete emptying, pain during defecation) or sexual function, including similar rates of dyspareunia (21.4% in the PAWG, 16.7% in the SPG). These outcomes may be influenced by uncontrolled variables such as diet, stool consistency, or insufficient PFM training intensity. Nevertheless, they support existing literature suggesting that PFM training affects not only urinary function but also bowel and sexual health, contributing to overall quality of life in female athletes [52,53].

A key strength of this study was its comprehensive assessment of pelvic floor function, encompassing urinary, bowel, and sexual dimensions. The inclusion of a control group and structured evaluation protocol further reinforce the validity of the findings. However, limitations must be acknowledged. The small sample size (*n* = 32) may have reduced statistical power, limiting the detection of significant differences. Future research should investigate how different types of physical activity affect pelvic floor health and evaluate the effectiveness of targeted interventions to prevent UI in athletes. Additionally, increasing awareness and reducing stigma around UI is crucial to promoting access to effective training programs and improving the quality of life for affected individuals.

## 5. Conclusions

The findings of this study highlight a notable prevalence of urinary incontinence (UI) among nulliparous female athletes, particularly soccer players, with a reported rate of 50%. The most common type identified was stress urinary incontinence (SUI), although severity was predominantly mild. In comparison, 35.7% of the control group—composed of physically active women—also reported UI, with SUI being the most prevalent form.

These results underscore the need to implement targeted pelvic floor muscle (PFM) strengthening and education programs for female athletes to effectively prevent and manage UI. Moreover, promoting pelvic floor health from a preventive standpoint may contribute to improved overall quality of life and potentially extend athletes’ sporting careers.

This study provides valuable evidence on the relationship between high-intensity physical activity and pelvic floor health, emphasizing the importance of incorporating preventive strategies within athletic environments to enhance both performance and well-being in female athletes.

## Figures and Tables

**Table 1:** Descriptive variables.

Variables	Soccer Players Group (*n* = 18)		Active Women Group (*n* = 14)		
Demographic Data	Mean ± SD	95% CI	Mean ± SD	95% CI	*p*-Value
Age (years)	26 ± 5.6	(23.1–28.7)	30.6 ± 5.00	(27.7–30.6)	≤0.05
Weight (kg)	62.2 ± 6.5	(58.9–65.4)	64.7 ± 9.2	(59.4–70)	0.37
BMI (kg/m^2^)	22.2 ± 2.1	(21.1–23.2)	23.9 ± 3.7	(21.8–26)	0.11
BFM (%)	12.2 ± 3.1	(10.7–13.7)	17.0 ± 8.6	(12–22)	0.1
Training session (per week)	6.1 ± 0.5	(5.9–6.4)	3.29 ± 1.3	(2.5–4.1)	≤0.001
Minutes per week	550	-	296.1	-	-

BMI—Body Mass Index; BFM—Body Fat Mass; SD—standard deviation; CI—confidence interval of mean; *n*—total participants.

**Table 2:** ICIQ-SF data analysis.

Variables	Soccer Players Group (*n* = 18)		Active Women Group (*n* = 14)		*p*-Value
ICIQ-Questionnaire	Mean ± SD		Mean ± SD		
ICIQ-SF Value	2.4 ± 3.0		3.0 ± 5.2		0.70
	*n*	%	*n*	%	% Total (*n* = 32)
UI prevalence	9	50	5	35.7	43.8
ICIQ-SF UI Severity	*n*	%	*n*	%	% Total (*n* = 32)
Mild UI (1–5 points)	7	77.8	1	20.0	25.0
Moderate UI (6–12 points)	2	22.3	3	60.0	15.6
Severe UI (13–18 points)	-	-	1	20.0	3.1
Very severe UI (up to 18 points)	-	-	-	-	-
UI type	*n*	%	*n*	%	% Total (*n* = 32)
SUI	9	100	3	60.0	37.5
UUI	-	-	1	20.0	3.1
MUI	-	-	1	20.0	3.1

SD—standard deviation; *n*—total participants; ICIQ-SF—International Consultation on Incontinence Questionnaire—Short Form; UI—urinary incontinence; SUI—stress urinary incontinence; UUI—urgency urinary incontinence; MUI—mixed urinary incontinence.

**Table 3:** Physical examination of PFM.

Variables	Soccer Players Group (*n* = 18)		Active Women Group (*n* = 14)		*p*-Value
	*n*	%	*n*	%	
Transverso contraction					0.96
yes	14	77.8%	11	78.6%	
no	4	22.2%	3	21.4%	
PFM contraction					0.96
yes	14	77.8%	11	78.6%	
no	4	22.2%	3	21.4%	
Endurance +15″					0.46
yes	6	33.3%	3	21.4%	
no	12	66.7%	11	78.6%	
Relaxation Capacity					0.85
yes	11	61.1%	9	64.3%	
no	7	38.9%	5	35.7%	
Involuntary PFM contraction					0.37
yes	1	3.1%	0	-	
no	17	96.9%	14	100%	
KNACK					0.2
yes	5	27.8%	7	50%	
no	13	72.2%	7	50%	
Fast contraction 60″					0.69
yes	9	50%	8	57.1%	
no	9	50%	6	42.9%	
Dynamical voluntary contractions 20″ (repetitions)	mean ± SD		mean ± SD		0.37
	7.4 ± 5.9		9.4 ± 6.3		
PFM pressure generated (cmH_2_O)	mean ± SD		mean ± SD		0.96
	1.0 ± 1.4		1.5 ± 1.1		

SD—standard deviation; *n*—total participants; PFM—pelvic floor muscle contraction; KNACK—voluntary pre-contraction of the pelvic floor before abdominal activation.

## Data Availability

Data are available upon request to the contact author.

## References

[1] Mäkiniemi J.K., Savolainen E.H., Finni T., Ihalainen J.K. (2023). Position Specific Physical Demands in Different Phases of Competitive Matches in National Level Women’s Football. Biol. Sport.

[2] González J.R., Cáceres A., Ferrer E., Balagué-Dobón L., Escribà-Montagut X., Sarrat-González D., Quintás G., Rodas G. (2024). Predicting Injuries in Elite Female Football Players With Global-Positioning-System and Multiomics Data. Int. J. Sports Physiol. Perform..

[3] Risberg M.A., Steffen K., Nilstad A., Myklebust G., Kristianslund E., Moltubakk M.M., Krosshaug T. (2018). Normative Quadriceps and Hamstring Muscle Strength Values for Female, Healthy, Elite Handball and Football Players. J. Strength. Cond. Res..

[4] Magaña-Ramírez M., Gallardo-Gómez D., Álvarez-Barbosa F., Corral-Pernía J.A. (2024). What Exercise Programme Is the Most Appropriate to Mitigate Anterior Cruciate Ligament Injury Risk in Football (Soccer) Players? A Systematic Review and Network Meta-Analysis. J. Sci. Med. Sport.

[5] Sebastian-Rico J.M., Muñoz-Fernández M.J., Martínez-Aranda L.M., Calvo-Lluch Á., Ortega-Becerra M. (2024). Prevalence of Urinary Incontinence in Female Professional Soccer Players by Category and Specific Position: A Comparative Study with a Control Group. Healthcare.

[6] Donnelly G.M., Moore I.S. (2023). Sports Medicine and the Pelvic Floor. Curr. Sports Med. Rep..

[7] Leitner M., Moser H., Eichelberger P., Kuhn A., Radlinger L. (2017). Evaluation of Pelvic Floor Muscle Activity during Running in Continent and Incontinent Women: An Exploratory Study. Neurourol. Urodyn..

[8] Leitner M., Moser H., Eichelberger P., Kuhn A., Baeyens J.-P., Radlinger L. (2018). Evaluation of Pelvic Floor Kinematics in Continent and Incontinent Women during Running: An Exploratory Study. Neurourol. Urodyn..

[9] Moser H., Leitner M., Eichelberger P., Kuhn A., Baeyens J.-P., Radlinger L. (2019). Pelvic Floor Muscle Displacement during Jumps in Continent and Incontinent Women: An Exploratory Study. Neurourol. Urodyn..

[10] Koenig I., Eichelberger P., Luginbuehl H., Kuhn A., Lehmann C., Taeymans J., Radlinger L. (2021). Activation Patterns of Pelvic Floor Muscles in Women with Incontinence While Running: A Randomized Controlled Trial. Int. Urogynecol. J..

[11] González-Ruiz de León C., Pérez-Haro M.L., Jalón-Monzón A., García-Rodríguez J. (2017). Female urinary incontinence: An update. Semergen.

[12] Cardoso A.M.B., Lima C.R.O.d.P., Ferreira C.W.S. (2018). Prevalence of Urinary Incontinence in High-Impact Sports Athletes and Their Association with Knowledge, Attitude and Practice about This Dysfunction. Eur. J. Sport Sci..

[13] de Mattos Lourenco T.R., Matsuoka P.K., Baracat E.C., Haddad J.M. (2018). Urinary Incontinence in Female Athletes: A Systematic Review. Int. Urogynecol. J..

[14] Fernandes A., Fitz F., Silva A., Filoni E., Filho J.M. (2014). Evaluation of the Prevalence of Urinary Incontinence Symptoms in Adolescent Female Soccer Players and Their Impact on Quality of Life. Occup. Environ. Med..

[15] Hagovska M., Švihra J., Buková A., Dračková D., Švihrová V. (2018). Prevalence and Risk of Sport Types to Stress Urinary Incontinence in Sportswomen: A Cross-Sectional Study. Neurourol. Urodyn..

[16] Culleton-Quinn E., Bø K., Fleming N., Cusack C., Daly D. (2024). Prevalence and Experience of Urinary Incontinence Among Elite Female Gaelic Sports Athletes. Int. Urogynecol. J..

[17] Da Roza T., Brandão S., Mascarenhas T., Jorge R.N., Duarte J.A. (2015). Urinary Incontinence and Levels of Regular Physical Exercise in Young Women. Int. J. Sports Med..

[18] Shaw J.M., Nygaard I.E. (2017). Role of Chronic Exercise on Pelvic Floor Support and Function. Curr. Opin. Urol..

[19] Bø K., Nygaard I.E. (2020). Is Physical Activity Good or Bad for the Female Pelvic Floor? A Narrative Review. Sports Med..

[20] Álvarez-García C., Doğanay M. (2022). The Prevalence of Urinary Incontinence in Female CrossFit Practitioners: A Systematic Review and Meta-Analysis. Arch. Esp. Urol..

[21] Crossley K.M., Patterson B.E., Culvenor A.G., Bruder A.M., Mosler A.B., Mentiplay B.F. (2020). Making Football Safer for Women: A Systematic Review and Meta-Analysis of Injury Prevention Programmes in 11,773 Female Football (Soccer) Players. Br. J. Sports Med..

[22] Gan Z.S., Smith A.L. (2023). Urinary Incontinence in Elite Female Athletes. Curr. Urol. Rep..

[23] Bø K. (2004). Urinary Incontinence, Pelvic Floor Dysfunction, Exercise and Sport. Sports Med..

[24] Bø K., Sherburn M. (2005). Evaluation of Female Pelvic-Floor Muscle Function and Strength. Phys. Ther..

[25] Coyne K.S., Sexton C.C., Irwin D.E., Kopp Z.S., Kelleher C.J., Milsom I. (2008). The Impact of Overactive Bladder, Incontinence and Other Lower Urinary Tract Symptoms on Quality of Life, Work Productivity, Sexuality and Emotional Well-Being in Men and Women: Results from the EPIC Study. BJU Int..

[26] Bo K., Frawley H.C., Haylen B.T., Abramov Y., Almeida F.G., Berghmans B., Bortolini M., Dumoulin C., Gomes M., McClurg D. (2017). An International Urogynecological Association (IUGA)/International Continence Society (ICS) Joint Report on the Terminology for the Conservative and Nonpharmacological Management of Female Pelvic Floor Dysfunction. Neurourol. Urodyn..

[27] Pires T., Pires P., Moreira H., Viana R. (2020). Prevalence of Urinary Incontinence in High-Impact Sport Athletes: A Systematic Review and Meta-Analysis. J. Hum. Kinet..

[28] Joseph C., Srivastava K., Ochuba O., Ruo S.W., Alkayyali T., Sandhu J.K., Waqar A., Jain A., Poudel S. (2021). Stress Urinary Incontinence Among Young Nulliparous Female Athletes. Cureus.

[29] Consejo de Europa (1997). Convenio Para La Protección de Los Derechos Humanos y La Dignidad Del Ser Humano Con Respecto a Las Aplicaciones de La Biología y La Medicina (Convenio Relativo a Los Derechos Humanos y a La Biomedicina). Boletín Oficial del Estado.

[30] Jefatura Del Estado Ley Orgánica 3/2018 3/2018, de 5 de Diciembre, de Protección de Datos Personales y Garantía de Los Derechos Digitales. Boletín Oficial del Estado 2018, No. 294, 119788–199857. https://www.boe.es/buscar/act.php?id=BOE-A-2018-16673.

[31] Alomía León R., Peña-Toncoso S., Hernández- Mosqueira C., Espinoza Cortez J. (2022). Comparación de los métodos de antropometría y bioimpedancia eléctrica a través de la determinación de la composición corporal en estudiantado universitario. MHSalud.

[32] Avery K., Donovan J., Peters T.J., Shaw C., Gotoh M., Abrams P. (2004). ICIQ: A Brief and Robust Measure for Evaluating the Symptoms and Impact of Urinary Incontinence. Neurourol. Urodyn..

[33] Espuña Pons M., Castro Díaz D., Carbonell C., Dilla T. (2007). Comparación Entre El Cuestionario “ICIQ-UI Short Form” y El “King’s Health Questionnaire” Como Instrumentos de Evaluación de La Incontinencia Urinaria En Mujeres. Actas Urológicas Españolas.

[34] Espuña Pons M., Rebollo Álvarez P., Puig Clota M. (2004). Validación de La Versión Española Del International Consultation on Incontinence Questionnaire-Short Form. Un Cuestionario Para Evaluar La Incontinencia Urinaria. Med. Clin..

[35] Vicente E., Barrio M., Gual J., Fadil Y., Capdevila M., Muñoz J., Garcia D., Hannaoui N., Prats J. (2016). Spanish (Spain) Validation of a Specific Symptomatic Questionnaire for Male Patients with Nocturia. Neurourol. Urodyn..

[36] Sisconeto de Freitas S., Cabral A.L., de Melo Costa Pinto R., Resende A.P.M., Pereira Baldon V.S. (2019). Effects of Perineal Preparation Techniques on Tissue Extensibility and Muscle Strength: A Pilot Study. Int. Urogynecol. J..

[37] da Silva J.B., de Godoi Fernandes J.G., Caracciolo B.R., Zanello S.C., de Oliveira Sato T., Driusso P. (2021). Reliability of the PERFECT Scheme Assessed by Unidigital and Bidigital Vaginal Palpation. Int. Urogynecol. J..

[38] Bø K. (2004). Pelvic Floor Muscle Training Is Effective in Treatment of Female Stress Urinary Incontinence, but How Does It Work?. Int. Urogynecol. J. Pelvic Floor Dysfunct..

[39] Messelink B., Benson T., Berghmans B., Bø K., Corcos J., Fowler C., Laycock J., Lim P.H.-C., van Lunsen R., á Nijeholt G.L. (2005). Standardization of Terminology of Pelvic Floor Muscle Function and Dysfunction: Report from the Pelvic Floor Clinical Assessment Group of the International Continence Society. Neurourol. Urodyn..

[40] Mendoza E.R., Dos Santos K.M., da Luz S.C.T., Da Roza T. (2021). Comparison of Urinary Incontinence, Based on Pelvic Floor and Abdominal Muscle Strength, between Nulliparous Female Athletes and Non-Athletes: A Secondary Analysis. Neurourol. Urodyn..

[41] Syeda F., Pandit U. (2024). Urinary Incontinence in Female Athletes: A Systematic Review on Prevalence and Physical Therapy Approaches. Cureus.

[42] Hagovska M., Švihra J., Buková A., Horbacz A., Dračková D., Švihrová V., Kraus L. (2017). Prevalence of Urinary Incontinence in Females Performing High-Impact Exercises. Int. J. Sports Med..

[43] Bø K., Sundgot-Borgen J. (2010). Are Former Female Elite Athletes More Likely to Experience Urinary Incontinence Later in Life than Non-Athletes?. Scand. J. Med. Sci. Sports.

[44] Almeida M.B.A., Barra A.A., Saltiel F., Silva-Filho A.L., Fonseca A.M.R.M., Figueiredo E.M. (2016). Urinary Incontinence and Other Pelvic Floor Dysfunctions in Female Athletes in Brazil: A Cross-Sectional Study. Scand. J. Med. Sci. Sports.

[45] Alves J.O., Luz S.T.D., Brandão S., Da Luz C.M., Jorge R.N., Da Roza T. (2017). Urinary Incontinence in Physically Active Young Women: Prevalence and Related Factors. Int. J. Sports Med..

[46] Goldstick O., Constantini N. (2014). Urinary Incontinence in Physically Active Women and Female Athletes. Br. J. Sports Med..

[47] Nygaard I., Shaw J., Egger M.J. (2012). Exploring the Association between Lifetime Physical Activity and Pelvic Floor Disorders: Study and Design Challenges. Contemp. Clin. Trials.

[48] Rodrigues M.P., Bérubé M.-È., Charette M., McLean L. (2024). Conservative Interventions for Female Exercise-Induced Urinary Incontinence: A Systematic Review. BJU Int..

[49] Parr R., Jones E., Figuers C., Ewen H.H. (2025). Relationship of Sport Variables on Stress Urinary Incontinence in Nulliparous Collegiate Athletes. J. Women Pelvic Health Phys. Ther..

[50] Barbosa-Silva J., Zanello S.C., Jorge C.H., Driusso P. (2024). Do Young Women Have an Accurate Perception about Their Pelvic Floor Muscle Contraction? An Agreement Study about Self-Perception and Physical Evaluation of the Pelvic Muscles Contraction by the PERFECT Scheme. J. Bodyw. Mov. Ther..

[51] Devreese A., Staes F., De Weerdt W., Feys H., Van Assche A., Penninckx F., Vereecken R. (2004). Clinical Evaluation of Pelvic Floor Muscle Function in Continent and Incontinent Women. Neurourol. Urodyn..

[52] Frigerio M., Barba M., Cola A., Braga A., Celardo A., Munno G.M., Schettino M.T., Vagnetti P., De Simone F., Di Lucia A. (2022). Quality of Life, Psychological Wellbeing, and Sexuality in Women with Urinary Incontinence-Where Are We Now: A Narrative Review. Medicina.

[53] Curillo-Aguirre C.A., Gea-Izquierdo E. (2023). Effectiveness of Pelvic Floor Muscle Training on Quality of Life in Women with Urinary Incontinence: A Systematic Review and Meta-Analysis. Medicina.

